# Protein palmitoylation in hematological malignancies: mechanistic links to lipid metabolic reprogramming and therapy resistance

**DOI:** 10.3389/fimmu.2026.1942607

**Published:** 2026-08-25

**Authors:** Qian Zhao, Jiangang Mei, Siqi Qian, Jingjing Pu, Feng Li

**Affiliations:** 1 Department of Hematology, Jinling Hospital, Affiliated Hospital of Medical School, Nanjing University, Nanjing, China; 2 Department of Hematology, Renji Hospital, Shanghai Jiao Tong University School of Medicine, Shanghai, China

**Keywords:** depalmitoylases, hematologic malignancies, lipid metabolic reprogramming, protein palmitoylation, therapeutic resistance, ZDHHC palmitoyltransferases

## Abstract

Protein palmitoylation has emerged as a central post-translational mechanism governing protein localization, stability, and signal transduction. In hematological malignancies, this dynamic modification lies at the intersection of lipid metabolic reprogramming, tumor–immune interactions, and therapeutic resistance. Here, we review how dysregulated palmitoyltransferases and depalmitoylases modify metabolic enzymes, lipid transporters, lipid-droplet proteins, oncogenic signaling components, and immune-regulatory molecules, thereby creating self-reinforcing circuits that sustain tumor growth and treatment failure. We further discuss the contribution of macrophage and myeloid-cell lipid metabolism, the potential roles of palmitoylation in non-malignant cells of the tumor microenvironment, and the underexplored relevance of palmitoylation to multiple myeloma. Particular attention is given to crosstalk with phosphorylation and ubiquitination, as well as to the distinction between direct evidence from hematological malignancies and cross-tumor mechanistic inferences. Finally, we evaluate emerging therapeutic strategies, including selective enzyme inhibitors, proteolysis-targeting chimeras, metabolic modulation, and immunotherapy combinations. This evidence-calibrated framework highlights both the translational potential of targeting palmitoylation and the need for cell-type-resolved validation in blood cancers.

## 1 Introduction

Protein palmitoylation is a reversible lipid modification that regulates protein membrane trafficking, stability, and signal transduction (1). Unlike other lipid modifications, palmitoylation is highly dynamic, enzymatically controlled by palmitoyl acyltransferases (PATs, mainly ZDHHCs) for addition and by depalmitoylases (e.g., APT1, PPT1) for removal (2). This cycling enables cells to rapidly respond to metabolic and environmental cues. Beyond its roles in neuronal and immune function (3, 4), palmitoylation has emerged as a key player in cancer. In hematological malignancies such as acute myeloid leukemia (AML), lymphomas, and multiple myeloma (MM), lipid metabolic reprogramming fuels proliferation, supports survival in the bone marrow niche, and drives therapy resistance (5–7).

Accumulating evidence now positions palmitoylation at the center of this metabolic transformation. It directly modifies enzymes in fatty acid synthesis and desaturation, lipid uptake receptors (CD36), and lipid droplet proteins (8–10), thereby enhancing *de novo* lipogenesis and lipid storage to support tumor growth (11). Concurrently, palmitoylation is essential for the membrane localization and oncogenic activity of drivers such as NRAS, FLT3-ITD, and β-catenin, integrating proliferative signaling with lipid anabolism (2, 12). This dual role not only sustains malignancy but also drives therapy resistance by modulating immune checkpoints and preserving leukemia-initiating cells responsible for relapse (13–17). Together, these interconnected axes establish palmitoylation as a central regulator of treatment failure in hematologic cancers.

Despite these advances, key challenges remain. First, the enzymatic complexity of palmitoylation hinders the development of selective inhibitors. Over 20 zinc finger DHHC-type palmitoyl acyltransferase (ZDHHC) enzymes exhibit overlapping substrate specificities (18, 19). Second, crosstalk between palmitoylation and other post-translational modifications (e.g., phosphorylation, ubiquitination) may explain adaptive resistance but remains poorly understood (20). Third, concerns over on-target toxicity in normal hematopoiesis and immune function create a significant translational gap (21, 22).

Notably, no systematic review has yet integrated the roles of palmitoylation in lipid metabolism and therapy resistance in hematologic malignancies. Here, we synthesize recent mechanistic discoveries, critically evaluate the roles of specific PATs and depalmitoylases, and assess emerging therapeutic strategies including small-molecule inhibitors, PROTACs, and combination regimens. By providing this integrated perspective, we aim to guide the development of palmitoylation-targeted interventions that overcome therapeutic resistance in hematologic cancers (2, 23, 24).

## 2 Palmitoylation biology & enzymatic regulation in hematologic malignancies

**Figure 1:**
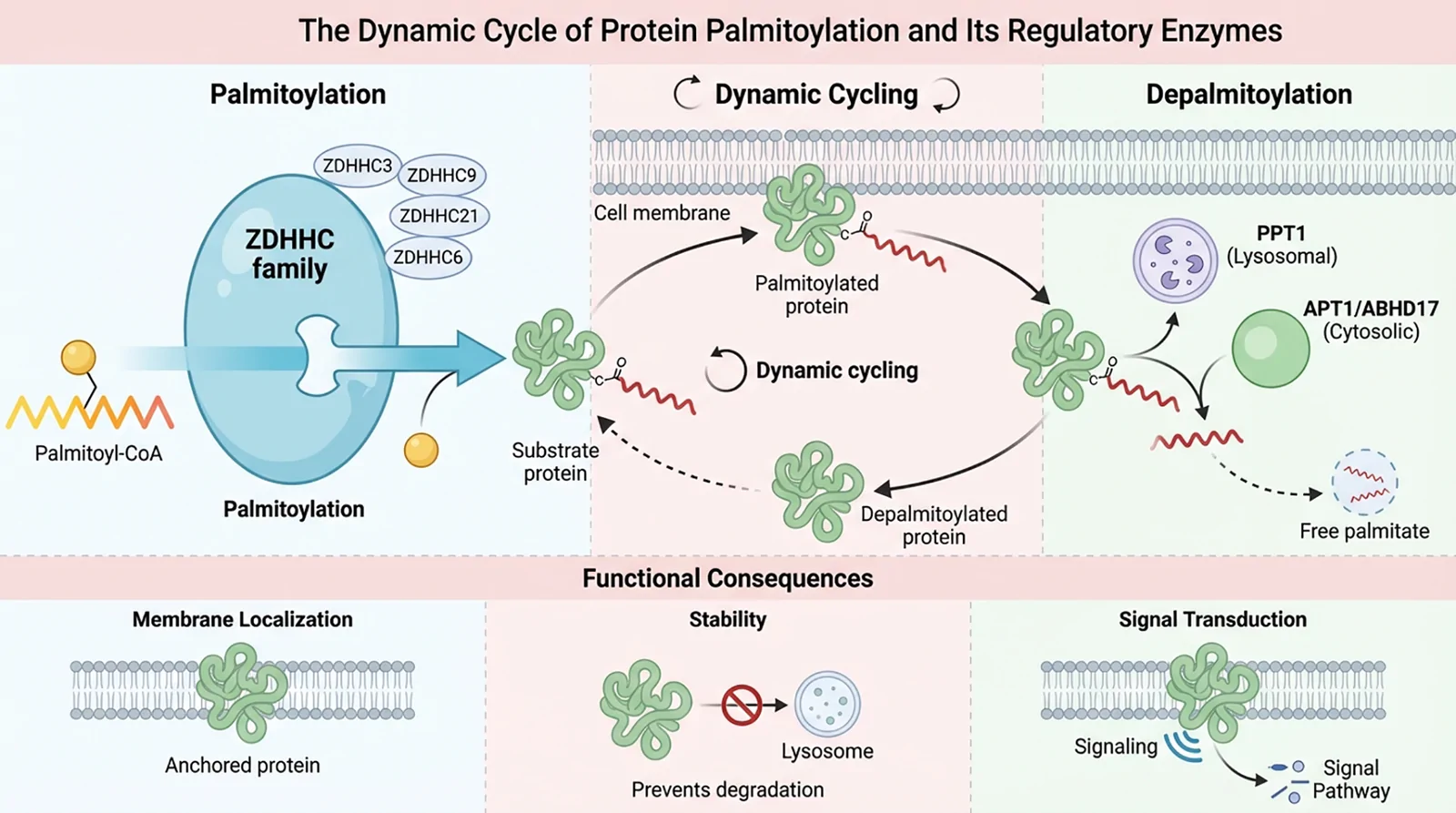
The dynamic cycle of protein palmitoylation and its regulatory enzymes. Protein palmitoylation is a reversible lipid modification catalyzed by ZDHHC family palmitoyl acyltransferases (e.g., ZDHHC3, ZDHHC6, ZDHHC9, ZDHHC21), which transfer palmitate from palmitoyl-CoA to cysteine residues of substrate proteins via a thioester bond. This modification is removed by depalmitoylating enzymes, including lysosomal PPT1 and cytosolic APT1/ABHD17 family members. The dynamic cycling between palmitoylation and depalmitoylation enables precise spatiotemporal control of protein membrane localization, stability, and signal transduction, thereby regulating diverse cellular processes. Illustration created by the authors.

Protein S-palmitoylation is a dynamic, reversible post-translational lipid modification. Unlike myristoylation or prenylation, palmitoylation’s reversibility allows for rapid cycling of proteins between membrane compartments, critically regulating their stability, trafficking, and function (25, 26). This cycling is enzymatically controlled by PATs, primarily the ZDHHC enzyme family for addition and by depalmitoylases (e.g., APT1/2, PPT1) for removal (**Figure 1**) (2). The addition of palmitate is catalyzed by a family of enzymes containing a conserved Asp-His-His-Cys (DHHC) motif, known as ZDHHC proteins. In mammals, 24 ZDHHC isoforms have been identified, each with potential substrate specificity and tissue expression patterns (1). In hematologic malignancies, the fine-tuned balance of this modification is profoundly disrupted. Aberrant expression and activity of these regulatory enzymes reprogram cellular signaling and metabolic networks, establishing a pro-tumorigenic state that supports cancer cell survival, proliferation, and adaptation to therapeutic stress (27, 28). This enzymatic imbalance sets the stage for the next layer of regulation, namely the functional reprogramming of lipid metabolism by palmitoylated target proteins.

### 2.1 Chemical dynamics and functional consequences of palmitoylation

The defining chemical feature of palmitoylation is the labile thioester bond, which facilitates rapid on-and-off cycling. This dynamic nature enables precise spatiotemporal control over protein localization, particularly directing modified proteins to lipid rafts, cholesterol- and sphingolipid-enriched plasma membrane microdomains that serve as hubs for signal transduction (25, 29). Molecular dynamics simulations confirm that palmitoylation alters protein-membrane interactions, favoring partitioning into ordered membrane phases crucial for effective signaling (29).

Functionally, this modification acts as a multifaceted regulatory switch. First, it governs protein membrane affinity and trafficking. For instance, palmitoylation of G protein subunits is essential for their stable plasma membrane association and downstream signaling initiation (25). Second, it modulates protein-protein interactions and stability. Palmitoylation can shield proteins from ubiquitination and degradation or facilitate their inclusion into specific signaling complexes (20). Third, it directly influences enzyme activity. The palmitoylation of key metabolic enzymes, such as fatty acid synthase (FASN), can enhance their dimerization and catalytic efficiency, thereby driving biosynthetic pathways and Chemoresistance (8). This broad functional impact emphasizes why the loss of palmitoylation homeostasis is detrimental in cancer. To probe these modifications directly, researchers have employed the removable backbone modification (RBM) strategy, which enables the chemical synthesis of homogeneously palmitoylated proteins for precise mechanistic studies (26).

### 2.2 Palmitoylation in hematological malignancies

Among hematologic malignancies, aberrant protein palmitoylation and its regulatory enzymes have been most intensively studied in AML, with a growing body of evidence also implicating its role in lymphomas. Notably, the functional impact of palmitoylation in these diseases is context-dependent, with specific ZDHHC enzymes targeting distinct oncogenic substrates to drive particular aspects of malignancy.

In AML, the dependency of key oncoproteins on palmitoylation for function is well-established. Experimental evidence demonstrates that palmitoylation is a critical regulator for the leukemogenic activity of both NRAS and the KRAS4A isoform (30, 31), mutant NRAS requires palmitoylation at cysteine 181 for membrane localization and leukemogenic transformation (12). Beyond RAS, recent research has identified specific palmitoyltransferases as critical nodes that control distinct oncogenic pathways. For instance, ZDHHC21 induces a differentiation block and maintains stemness in AML by regulating oxidative phosphorylation (15), linking palmitoylation directly to stem cell biology. ZDHHC3-mediated palmitoylation regulates the oncogenic transcriptional activity of PML/RARα in acute promyelocytic leukemia (APL) pathogenesis, with knockdown or overexpression of ZDHHC3 altering the expression of proliferation- and differentiation-related genes (32). Interestingly, ZDHHC6-mediated palmitoylation exerts a restraining effect on FLT3-ITD, limiting its surface expression and signaling, while rewiring downstream signaling through AKT and ERK pathways in addition to STAT5 (33). This example illustrates that palmitoylation can both activate and constrain oncogenic signaling depending on the context. Additionally, polydatin upregulates ZDHHC19 to promote apoptosis of AML cells via mediating BH3 interacting-domain death agonist palmitoylation (34), suggesting that therapeutic modulation of specific ZDHHC enzymes could have beneficial effects.

In lymphomas, inhibition of ZDHHC11 decreased growth of several B-cell lymphoma subtypes, including Burkitt, Hodgkin and diffuse large B-cell lymphoma (DLBCL) lines. Similarly, knockdown of ZDHHC18 in four Burkitt lymphoma cell lines reduced cell viability (35). In DLBCL, downregulation of palmitoyltransferase ZDHHC21 expression is associated with poor prognosis in patients. It reduces the stability of FASN by mediating its palmitoylation, so the absence of ZDHHC21 can lead to FASN stability and increased fatty acid synthesis, promoting lymphoma progression (36). A study demonstrated that inactivating GNA13 by targeting its palmitoylation enhanced the sensitivity of GCB-DLBCL to the BCL2 inhibitor (37). However, there are no research reports on palmitoylation in MM. Understanding these enzymatic drivers provides the rationale for developing targeted therapies aimed at restoring palmitoylation homeostasis in hematologic cancers (2, 18).

### 2.3 Multiple myeloma: an underexplored palmitoylation context

Direct mechanistic studies of protein palmitoylation in multiple myeloma (MM) remain scarce. Nevertheless, MM is characterized by extensive lipid metabolic dysregulation, dependence on endoplasmic-reticulum proteostasis, constitutive membrane-proximal signaling, and marked adaptation to the adipocyte-rich bone-marrow niche (6, 38). These features provide a strong biological rationale for investigating palmitoylation in this disease. By analogy with experimentally validated mechanisms in AML and lymphoma, palmitoylation could regulate the localization or stability of PI3K/AKT, RAS/MAPK, NF-κB, and unfolded-protein-response components; control CD36-dependent lipid uptake and lipid-droplet dynamics; and modulate immune-checkpoint abundance or resistance to proteasome inhibitors, immunomodulatory drugs, monoclonal antibodies, and T-cell–redirecting therapies. These possibilities are cross-tumor inferences rather than established MM mechanisms. Future studies should therefore combine palmitoyl-proteomics of primary plasma cells with CRISPR-based perturbation of candidate ZDHHC enzymes and depalmitoylases, paired diagnosis–relapse samples, and functional testing in bone-marrow–mimetic systems. Recent reviews of adoptive cellular immunotherapy in MM further underscore the expanding use of CAR-T, CAR-NK, CIK, and related immune-effector platforms, supporting the relevance of investigating palmitoylation-dependent regulation of immune escape and treatment response in this disease (39).

## 3 Palmitoylation-driven lipid metabolic reprogramming

**Figure 2:**
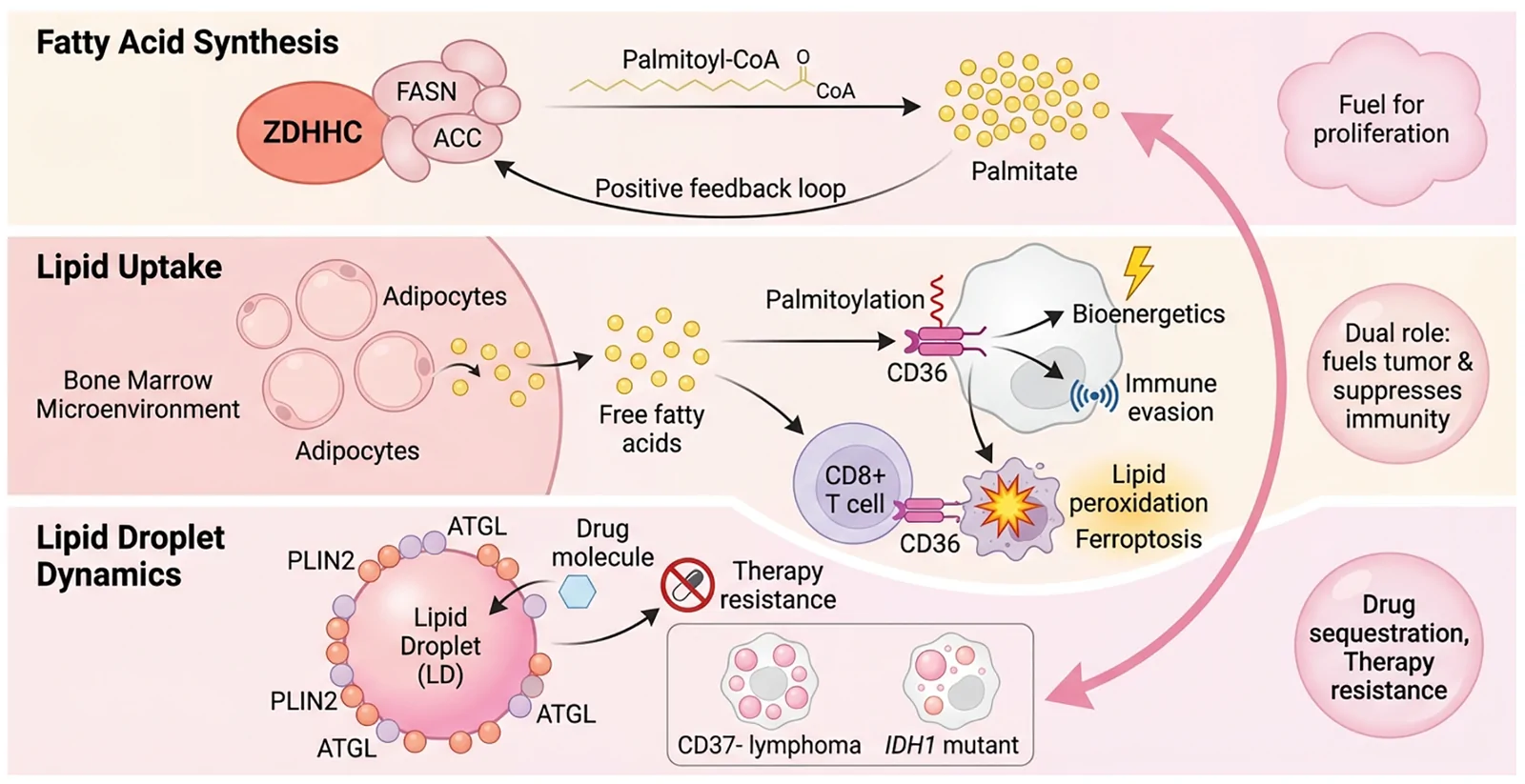
Palmitoylation-driven lipid metabolic reprogramming in hematologic malignancies. Palmitoylation directly modulates three interconnected aspects of lipid metabolism to support tumor growth and therapy resistance. (1) Fatty acid synthesis: Palmitoylation enhances the stability and activity of FASN and ACC, driving *de novo* palmitate production and creating a positive feedback loop. (2) Lipid uptake: Palmitoylation of CD36 promotes fatty acid import from the bone marrow niche. In tumor cells, this fuels immune evasion; in CD8+ T cells, CD36-mediated uptake induces lipid peroxidation and ferroptosis, impairing antitumor immunity. (3) Lipid droplet dynamics: PLIN2 and ATGL are regulated by palmitoylation, influencing lipid storage and mobilization. Lipid droplets can sequester lipophilic drugs, directly contributing to therapy resistance. Illustration created by the authors.

The dysregulated expression of ZDHHC enzymes documented above directly impacts the palmitoylation status of proteins that govern lipid metabolism. This section details how palmitoylation coordinately enhances *de novo* fatty acid synthesis, scavenges exogenous lipids, and integrates these metabolic fluxes with core oncogenic pathways to fuel tumorigenesis (**Figure 2**).

### 3.1 Regulation of fatty acid synthesis and desaturation

*De novo* fatty acid synthesis is a cornerstone of lipid reprogramming in cancer. Palmitoylation directly targets and modulates the key enzymes in this pathway, creating a self-amplifying metabolic loop. The core lipogenic enzymes, FASN and acetyl-CoA carboxylase (ACC), are regulated by palmitoylation. This modification enhances their stability, promotes membrane association, and increases enzymatic activity, thereby driving the production of palmitate, the foundational 16-carbon fatty acid (8, 11). Notably, this newly synthesized palmitate is not only a structural lipid but also a substrate for further palmitoylation reactions. This establishes a positive feedback loop. Increased lipogenesis provides more palmitoyl-CoA for protein modification, which in turn can further stabilize and activate lipogenic enzymes. Thus, palmitoylation creates a self-sustaining cycle that amplifies its own metabolic output. Beyond its role in cancer cells themselves, palmitoylation-mediated lipid metabolism also influences the tumor microenvironment. In multiple myeloma, palmitic acid has been identified as a factor that inhibits macrophage-mediated resistance to bortezomib and melphalan (38). This example illustrates that palmitoylation-dependent lipid metabolism can modulate both tumor cell-intrinsic properties and the surrounding microenvironment. Beyond synthesis, fatty acid desaturation intersects with protein palmitoylation at the level of substrate availability. Stearoyl-CoA desaturase 1 (SCD1) converts palmitate to palmitoleate, a monounsaturated fatty acid that serves as a lipid donor for the palmitoylation of specific proteins. Studies in murine models have demonstrated that SCD1 deficiency leads to palmitoleate depletion, which in turn impairs Wnt3a palmitoleoylation, a variant of palmitoylation, and disrupts Wnt/β-catenin signaling (40). While this mechanism has not yet been investigated in hematologic malignancies, it suggests that SCD1 may influence oncogenic signaling through its role in providing palmitoylation substrates.

### 3.2 Mediation of lipid uptake and lipid droplet dynamics

Tumor cells complement *de novo* synthesis by avidly scavenging lipids from their microenvironment, and palmitoylation governs both the uptake and storage of these lipids. The fatty acid translocase CD36 is a major gateway for exogenous lipid uptake. Its function is modulated by palmitoylation, which affects CD36 clustering at the plasma membrane and its endocytic recycling, thereby enhancing fatty acid import (10). This is particularly significant in the bone marrow, an adipocyte-rich niche that provides a ready supply of lipids. CD36 on AML cells senses oxidized low-density lipoprotein (OxLDL) to prime the TLR4-LYN-MYD88-nuclear factor κB (NF-κB) pathway. Exogenous palmitate transfer via CD36 further potentiates this innate immune pathway by supporting ZDHHC6-mediated MYD88 palmitoylation, ultimately promoting immune evasion in AML (41). CD36-mediated uptake of fatty acids by tumor-infiltrating CD8^+^ T cells in the tumor microenvironment induces lipid peroxidation and ferroptosis, leading to reduced cytotoxic cytokine production and impaired antitumor ability (42). Thus, palmitoylation-dependent lipid uptake exerts a dual role in the tumor microenvironment, fueling tumor growth while simultaneously suppressing anti-tumor immunity.

The accumulation of lipid droplets (LDs) is the hallmark of various human diseases, including atherosclerosis, nonalcoholic fatty liver disease, obesity and cancer. Palmitoylation regulates the key proteins governing LD biology. The coating protein perilipin 2 (PLIN2) and the lipase adipose triglyceride lipase (ATGL) are both subject to this modification. S-acylation of ATGL is required for lipid droplet homoeostasis in hepatocytes. A study showed that the post-translational S-acylation of ATGL at Cys15 by the zDHHC11^+^(B) acyltransferase has a crucial role in LD catabolism by enhancing the catalytic activity of ATGL (43). In blood cancers, a shift towards LD accumulation is common, driven by these modifications. LDs act not only as energy reserves but also as sinks for lipophilic drugs, contributing directly to therapy resistance (44). However, in hematological tumors, there are few studies on the pathogenesis mediated by lipid droplet palmitoylation. In aggressive B-cell lymphoma, tetraspanin CD37, a prognostic marker is essential membrane-localized inhibitor of fatty acid (FA) metabolism. Deletion of CD37 on lymphoma cells induces uptake and processing of exogenous palmitate into energy and essential building blocks for proliferation, meanwhile, results in increased FA oxidation. Large lipid deposits and intracellular lipid droplets are observed in CD37-negative lymphoma tissues of patients (45). Another study has shown that there is a significant accumulation of cytoplasmic LD in the original cells of B-cell acute lymphoblastic leukemia (B-ALL) patients with isocitrate dehydrogenase 1 (IDH1) mutations, while such accumulation is extremely rare in patients with IDH1 wild-type. This is a previously unrecognized morphological marker of this subtype. The experiments further confirmed that in B-ALL cell lines, IDH1 mutations or their carcinogenic metabolite R-2HG can directly induce LD formation and triglyceride biosynthesis. Moreover, the IDH1 mutation inhibitor AG-120 (Avelumab) can reverse this phenotype (46).

### 3.3 Integration with oncogenic signaling pathways

Palmitoylation does not operate in isolation; rather, it functions as a critical integrator that physically and functionally couples oncogenic signaling with lipid metabolism. This integration creates a vicious cycle: oncogenic signals demand more lipids for membrane biosynthesis and signaling, while enhanced lipid metabolism provides both the raw materials and the energetic substrate to sustain these signals.

Regulation of RAS/MAPK and PI3K/AKT signaling pathways: Palmitoylation is a crucial post-translational modification that regulates the subcellular localization and functional activation of RAS proteins. In hematologic malignancies, palmitoylation of NRAS and KRAS is required for their targeting to the plasma membrane and subsequent activation of downstream MAPK and PI3K/AKT signaling pathways (47). Evidence suggests that targeting the dynamic cycle of palmitoylation and depalmitoylation represents a promising therapeutic strategy for cancers harboring NRAS mutations (12). In disease-relevant *in vivo* models, disruption of N-RasG12D palmitoylation via the C181S mutation prevents its plasma membrane localization, reduces Raf/MEK/ERK signaling, alters hematopoietic stem and progenitor cell populations, and effectively suppresses NrasG12D-driven myeloid transformation (12), demonstrating the therapeutic potential of modulating palmitoylation in NRAS-mutated cancers. Mechanistically, the palmitoyltransferase ZDHHC9 has been identified as a key enzyme responsible for NRAS palmitoylation. In hematopoietic malignancies, upregulation of RAB27B resulting from CBL loss and JAK2 activation promotes the recruitment of ZDHHC9, thereby facilitating NRAS palmitoylation and plasma membrane localization (47). This finding suggests that targeting RAB27B may offer a novel therapeutic approach for NRAS-driven cancers. Beyond RAS itself, key components of the PI3K/AKT pathway are also regulated by palmitoylation. For example, palmitoylation of the PI3K regulatory subunit p85 and the effector AKT influences their membrane recruitment and activation, contributing to cancer cell survival (48). Although direct evidence primarily derives from solid tumor studies, the central role of the PI3K/AKT pathway in blood cancers implies that analogous regulatory mechanisms are likely operative. Moreover, multiple publicly available datasets were employed to conduct pan-cancer analysis, examining the transcriptome, genomic alterations, clinical outcomes, and correlation with c-Myc (Myc) for palmitoylation-related genes. It discovered that abnormal DNA methylation and oncogenic Myc-driven transcriptional regulation synergistically contribute to the dysregulation of palmitoylation-related genes. Through mass spectrometry and enrichment analyses, it also identified palmitoyl-acyltransferases ZDHHC7 and ZDHHC23 as significant contributors to mTOR signaling, DNA repair, and immune pathways, highlighting their potential roles in tumorigenesis (28).

**Figure 3:**
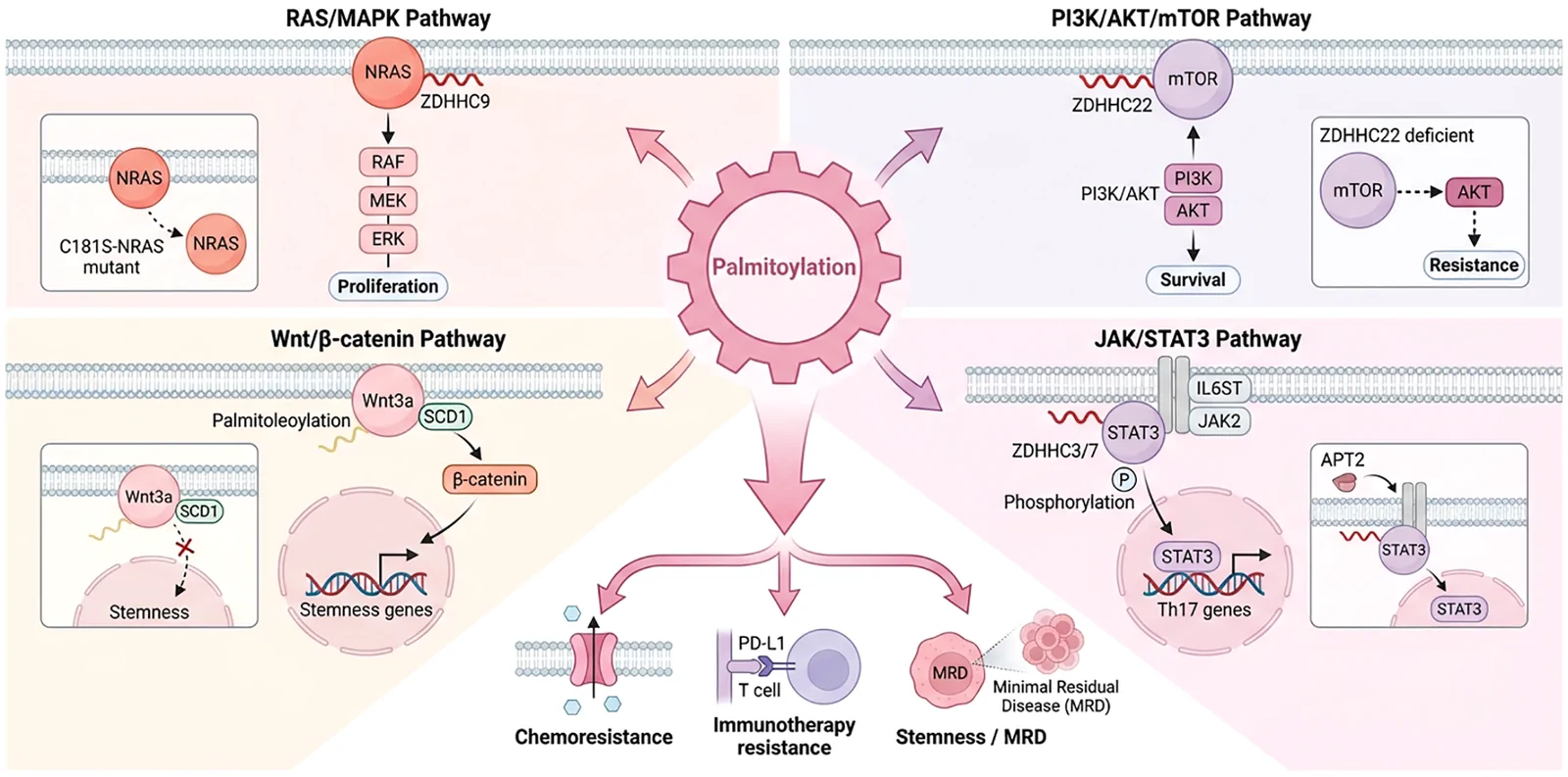
Palmitoylation integrates oncogenic signaling pathways with metabolic adaptation to drive therapy resistance. Palmitoylation acts as a central hub coupling multiple oncogenic pathways to lipid metabolic reprogramming. (1) RAS/MAPK pathway: ZDHHC9-mediated NRAS palmitoylation is essential for its membrane localization and downstream signaling; the C181S mutation disrupts this process. (2) PI3K/AKT/mTOR pathway: ZDHHC22-mediated mTOR palmitoylation regulates its stability; loss of this modification enhances AKT signaling and confers resistance. (3) Wnt/β-catenin pathway: SCD1-dependent palmitoleoylation of Wnt3a is required for β-catenin activation and stemness gene expression. (4) JAK/STAT3 pathway: ZDHHC3/7-mediated STAT3 palmitoylation promotes its membrane localization and phosphorylation; APT2-mediated depalmitoylation allows nuclear translocation. Together, these interconnected pathways converge to establish chemoresistance, immunotherapy resistance, and stemness-driven minimal residual disease (MRD). Illustration created by the authors.

Palmitoylation also exerts profound regulatory effects on the Wnt/β-catenin and JAK/STAT signaling pathways, both of which play critical roles in hematologic malignancies. Acyl-CoA synthetase long chain family member 5 (ACSL5), is a member of the acyl-CoA synthetases (ACSs) family that activates long chain fatty acids by catalyzing the synthesis of fatty acyl-CoAs. The expression of ACSL5 was higher in bone marrow cells from AML patients compared with healthy donors. ACSL5 level could serve as an independent prognostic predictor of the overall survival of AML patients. In AML cells, the knockdown of ACSL5 suppressed the activation of the Wnt/β-catenin pathway and inhibited cell growth both *in vitro* and *in vivo* by suppressing the palmitoylation modification of Wnt3a (49). Most studies on palmitoylation of the JAK/STAT pathway are related to autoimmune diseases. STAT3 itself is regulated by a palmitoylation-depalmitoylation cycle. ZDHHC7 and ZDHHC3 palmitoylate STAT3 at Cys108, promoting its plasma membrane localization where IL6ST and JAK2 are localized, thereby facilitating STAT3 phosphorylation. The phosphorylated STAT3 is then depalmitoylated by APT2, allowing it to translocate to the nucleus and induce genes important for Th17 cell differentiation (50). Thus, palmitoylation acts as a central integrator, coupling multiple oncogenic signaling pathways directly to the metabolic machinery that fuels tumor growth. This self-amplifying loop, in which oncogenic signals drive lipid metabolism and enhanced lipid metabolism sustains these signals, establishes a foundation for the development of therapy resistance (**Figure 3**).

### 3.4 Immune and stromal lipid metabolism in the tumor microenvironment

Lipid metabolic reprogramming in hematological malignancies extends beyond malignant cells. Tumor-associated macrophages (TAMs) can increase fatty-acid uptake, lipid-droplet storage, cholesterol handling, and mitochondrial fatty-acid oxidation in response to tumor-derived lipids and cytokines. These adaptations influence inflammatory signaling, efferocytosis, antigen presentation, reactive-oxygen-species production, and the balance between antitumor and immunosuppressive macrophage states. In a mechanistically informative non-cancer model, increased macrophage angiotensin-converting enzyme enhanced PPARα expression, mitochondrial import of long-chain fatty acids, β-oxidation, ATP production, cholesterol efflux, and efferocytosis (51). Although this study was conducted in atherosclerosis, it demonstrates how PPARα-associated lipid remodeling can fundamentally reshape macrophage function and provides a testable framework for lipid-rich bone-marrow and lymphoid niches.

Palmitoylation may add a spatial regulatory layer to these metabolic programs by controlling the membrane association, trafficking, and stability of inflammatory receptors, adaptors, lipid transporters, and mitochondrial proteins (1, 52). In TAMs and myeloid-derived suppressor cells (MDSCs), candidate consequences include altered TLR, STING, NF-κB, and inflammasome signaling; changes in CD36-dependent lipid handling; and modulation of cytokine production and suppressive activity. In stromal and cancer-associated fibroblast-like cells, palmitoylation may regulate adhesion receptors, chemokine secretion, extracellular-matrix interactions, and trophic support for malignant cells. Direct cell-type-resolved evidence in hematological malignancies is still limited; therefore, these mechanisms are presented as priority research gaps rather than established conclusions.

## 4 Mechanisms of therapy resistance

Therapeutic resistance remains the principal obstacle to curing hematologic malignancies. Protein palmitoylation has emerged as a critical, multifaceted driver of this resistance, enabling tumor cells to evade the cytotoxic effects of chemotherapy and targeted agents, suppress anti-tumor immunity. This section delineates the specific molecular mechanisms by which palmitoylation confers these distinct yet interconnected forms of treatment failure.

### 4.1 Promoting resistance to chemotherapy and targeted therapy

Palmitoylation is crucial for the membrane localization and activity of key survival kinases. For example, the palmitoylation of mTOR by ZDHHC22 normally limits its stability; loss of this modification leads to enhanced mTOR/AKT signaling and confers resistance to agents like tamoxifen (53). In the context of targeted therapy, palmitoylation facilitates adaptive resistance. In B-cell lymphomas treated with Bruton’s tyrosine kinase (BTK) inhibitors, palmitoylation of alternative kinases (e.g., SRC family kinases) can maintain downstream survival signals, allowing cells to bypass the inhibited primary target (18). This is epitomized in APL, where the palmitoylation of the PML/RARα oncoprotein by ZDHHC3 is essential for its leukemogenic activity; targeting ZDHHC3 can overcome resistance to standard all-trans retinoic acid (ATRA) and arsenic trioxide therapy (32). It also contributes to the stabilization of resistance promoting proteins; for instance, palmitoylation of cell death regulators like solute carrier family 7 membrane 11 (SLC7A11) prevents their degradation, resulting in therapeutic resistance (54, 55). Furthermore, palmitoylation reshapes oncogenic signaling by activating pro-survival pathways. As observed in sorafenib-resistant hepatocellular carcinoma, palmitoylation of the protein convertase subtilisin/kexin type 9 (PCSK9) enhances the activation of the phosphatidylinositol 3-kinase/protein kinase B (PI3K/AKT) pathway (56). From a clinical perspective, S-palmitoylation is implicated in resistance to multiple therapeutic modalities, highlighting its role as a key regulatory axis in treatment adaptation. Importantly, unlike resistance mechanisms driven by irreversible genetic alterations, palmitoylation-mediated resistance may be reversible through pharmacological intervention. In preclinical models, inhibitors targeting PATs such as ZDHHC3/9/20, or depalmitoylating enzymes (APTs) like PPT1, have demonstrated potent efficacy both as monotherapies and in combination with standard treatments (57–59).

### 4.2 Mediating immunotherapy resistance and immune escape

S-Palmitoylation exerts a dual influence on therapeutic resistance in cancer. Mechanistically, it regulates the subcellular localization of drug targets, allowing certain membrane-associated proteins (e.g., programmed death-ligand 1, PD-L1) to evade inhibition by targeted therapies (13, 14). S-palmitoylation is associated with reduced efficacy of anti-programmed cell death protein 1 (PD-1)/PD-L1 therapy. In 2019, two independent studies identified that both ZDHHC3 and ZDHHC9 catalyze PD-L1 palmitoylation at Cys272 (13, 14). This post-translational modification impairs PD-L1 lysosomal degradation, enhancing its stability and membrane localization, which ultimately allows tumor cells to evade immune surveillance by inhibiting T-cell activity. Disrupting PD-L1 palmitoylation and expression via ZDHHC knockout, pharmacological inhibition, or targeted degradation restores antitumor immune responses. This ZDHHC9/PD-L1 regulatory mechanism was later validated in non-small cell lung cancer (60). Further mechanistic clarification came in 2021, when Yao et al. reported that ZDHHC9 also mediates PD-1 palmitoylation at Cys912, promoting its trafficking to recycling endosomes and preventing its lysosomal degradation (61). Importantly, PD-1 palmitoylation, rather than that of PD-L1, was shown to be essential for activating mTOR signaling and driving therapeutic resistance in tumor cells. Beyond the PD-1/PD-L1 axis, palmitoylation also regulates the function of other immune checkpoint molecules such as B7-H4 and TIM-3. Studies have shown that ZDHHC3 palmitoylates B7-H4, inhibiting its lysosomal degradation and thereby sustaining tumor immunosuppression (16). Similarly, TIM-3 can be palmitoylated by ZDHHC3 at Cys9, a modification that promotes its interaction with sortilin and targets it for lysosomal degradation. The downregulation of TIM-3 resulting from this process is correlated with CD4^+^T-cell dysfunction (62). These findings highlight the dual role of palmitoylation in finely tuning antitumor immune responses by modulating the stability of immune checkpoint proteins. While anti-PD-1 therapy has shown efficacy in relapsed/refractory classic Hodgkin lymphoma (cHL) and non-Hodgkin lymphoma (NHL) (63–65), a subset of patients still experience primary resistance or disease progression after treatment. Currently, the mechanisms underlying PD-1 palmitoylation-mediated resistance in lymphoma remain unreported and warrant further investigation.

Beyond checkpoint stabilization on malignant cells, palmitoylation can directly shape immune-cell function. Reversible S-acylation controls the membrane residence and assembly of signaling complexes, and may thereby affect macrophage activation, mitochondrial organization, lipid uptake, cholesterol trafficking, and inflammatory output (1, 20, 52). These processes are relevant to TAMs and MDSCs, in which metabolic adaptation and inflammatory signaling jointly determine immunosuppressive activity. Palmitoylation may also influence T-cell receptor organization, co-stimulatory signaling, and metabolic fitness, underscoring a potential therapeutic trade-off: systemic inhibition could reduce tumor immune escape but also impair effector immune cells. Because most detailed mechanisms have been defined outside hematological tumors, future studies should distinguish tumor-cell and immune-cell dependencies using lineage-restricted perturbation and cell-type-resolved palmitoyl-proteomics. More broadly, contemporary analyses of T-cell biology in cancer emphasize that receptor organization, activation, exhaustion, and metabolic fitness jointly determine antitumor efficacy, providing a conceptual basis for studying palmitoylation as a regulator of T-cell function (66).

### 4.3 Maintaining stemness and minimal residual disease

The persistence of leukemia stem cells (LSCs) is responsible for relapse and minimal residual disease (MRD). LSCs rely on a unique metabolic and signaling state that is critically maintained by palmitoylation. Palmitoylation directly modifies and stabilizes core proteins that govern the stem cell state. For example, palmitoylation of β-catenin enhances its nuclear localization and transcriptional activity within the Wnt pathway, which is essential for LSC self-renewal (2). Notably, this regulatory axis extends to mitochondrial metabolism, a key vulnerability of LSCs. Recent studies have shown that the palmitoyltransferase ZDHHC21 critically maintains the oxidative phosphorylation (OXPHOS) hyperactivity in AML cells, especially in FLT3-ITD-mutated subtypes, by palmitoylating mitochondrial adenylate kinase 2 (AK2). Inhibition of ZDHHC21 disrupts OXPHOS, impairs LSC stemness, induces differentiation, and synergizes with chemotherapy, highlighting it as a promising therapeutic target to overcome relapse and treatment resistance in AML (15). Moreover, LSCs reside in protective bone marrow niches where stromal cells, particularly adipocytes, provide fatty acids (67). Palmitoylation has been shown to dynamically regulate CD36-mediated fatty acid uptake by controlling its membrane localization and endocytic recycling (10). This mechanism likely enables LSCs to efficiently utilize exogenous lipids from the niche to support their bioenergetic and biosynthetic needs. Indeed, a recent study in AML demonstrates that CD36-dependent lipid metabolic reprogramming promotes immune evasion and resistance to hypomethylating agents, underscoring the clinical relevance of this pathway (41). Furthermore, CD36-mediated lipid metabolism can dampen CD8^+^ T cell effector function via ferroptosis, suggesting additional layers of immune modulation (42). Additionally, inducing ferroptosis, a form of cell death triggered by lipid peroxidation, by targeting the altered lipid metabolism of LSCs is an active area of investigation (68). Ferroptosis has emerged as a critical cell death pathway in hematological malignancies, with studies across leukemia, lymphoma, and myeloma demonstrating its regulation by core lipid metabolism genes such as ACSL4 and glutathione peroxidase 4 (GPX4) (69). Recent studies have uncovered a novel regulatory mechanism: ZDHHC8-mediated palmitoylation of GPX4, which stabilizes GPX4 protein and sustains its activity in suppressing lipid peroxidation, thereby determining ferroptosis sensitivity (70, 71). Moreover, disruption of GPX4 palmitoylation has been shown to enhance anti-tumor immune responses (70), suggesting that this regulatory axis may represent an important link between protein lipidation, ferroptosis, and immune surveillance, with potential implications for hematological malignancies.

## 5 Therapeutic targeting strategies

**Figure 4:**
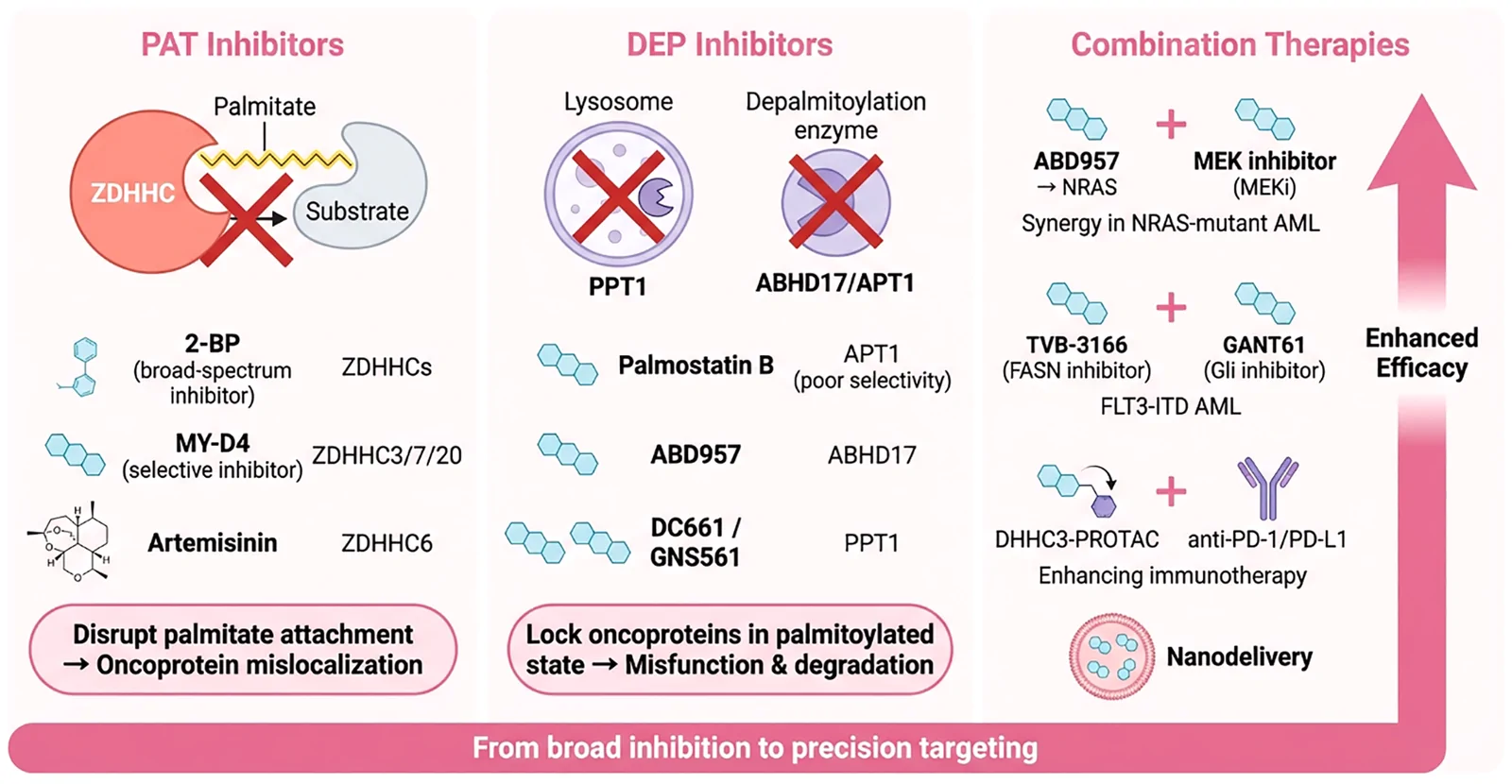
Therapeutic strategies targeting protein palmitoylation in hematologic malignancies. Three classes of therapeutic approaches have been developed to target the palmitoylation cycle. (1) PAT inhibitors: Broad-spectrum inhibitor 2-BP and selective inhibitors (MY-D4 targeting ZDHHC3/7/20, artemisinin targeting ZDHHC6) disrupt palmitate attachment, leading to oncoprotein mislocalization and inactivation. (2) DEP inhibitors: Palmostatin B targets APT1 but lacks selectivity; ABD957 selectively inhibits ABHD17 and synergizes with MEK inhibition in NRAS-mutant AML; DC661 and GNS561 target PPT1 and are under clinical evaluation. (3) Combination strategies: ABD957 plus MEK inhibitors, TVB-3166 (FASN inhibitor) plus GANT61, and DHHC3-PROTAC plus immune checkpoint blockade have shown synergistic efficacy in preclinical models. Nanotechnology-based platforms are also being developed to co-deliver such combinations. The field is moving from broad inhibition toward precision targeting of specific ZDHHC family members. Illustration created by the authors.

Given the central role of palmitoylation in driving the pathogenesis and treatment resistance of hematologic malignancies, targeting this modification and its regulatory enzymes has emerged as a promising therapeutic frontier. This section critically evaluates the current pharmacological strategies, focusing on inhibitors of palmitoyl acyltransferases (PATs) and depalmitoylases (DEPs), and explores the rationale for synergistic combination therapies designed to overcome the inherent adaptability of cancer cells (**Figure 4**).

### 5.1 Palmitoyl acyltransferase inhibitors

The development of agents targeting the ZDHHC family of PATs aims to disrupt the initial palmitate attachment, thereby mislocalizing and inactivating a broad spectrum of oncogenic client proteins. The prototypical PAT inhibitor 2-bromopalmitate (2-BP) has demonstrated proof-of-concept by globally suppressing protein palmitoylation and exhibiting antitumor activity in preclinical models (72). In B-cell lymphoma, 2-BP treatment induced apoptosis and suppressed cell growth, supporting the therapeutic potential of PAT inhibition in hematologic malignancies (35). However, 2-BP’s clinical utility is severely limited by poor selectivity, off-target effects, and systemic toxicity, highlighting the need for more precise agents (18). Recent efforts have focused on developing more selective inhibitors. For instance, MY-D4 has been identified as a selective inhibitor of ZDHHC3/7/20, capable of reducing substrate palmitoylation in cellular models (73). Additionally, artemisinin has been shown to function as a non-lipid inhibitor of ZDHHC6, directly targeting this palmitoyltransferase to reduce NRAS palmitoylation and downstream signaling (74). The critical role of specific ZDHHC enzymes in hematologic malignancies further underscores the need for isoform-selective targeting. Notably, DHHC3 has been identified as essential for the oncogenic activity of the PML/RARα fusion protein in APL, with genetic or pharmacological inhibition of DHHC3 suppressing leukemogenesis and overcoming resistance to standard therapies (32). This establishes DHHC3 as a compelling therapeutic target in APL and potentially other hematologic cancers driven by palmitoylation-dependent oncoproteins. Beyond conventional small-molecule inhibitors, cyclic PROTACs designed to selectively degrade DHHC3 have shown potent downregulation of PD-L1 and enhanced antitumor immunity in cervical cancer models, offering a novel strategy for achieving therapeutic specificity within the highly homologous ZDHHC family (23). Similarly, PROTAC-mediated degradation of ZDHHC5 and ZDHHC20 has been shown to decrease substrate palmitoylation and impair tumor cell function (24). The structural conservation among ZDHHC enzymes and their integral membrane nature pose substantial challenges for traditional small-molecule drug design, and the field is actively utilizing high-throughput screening, structure-based design, and innovative modalities like PROTACs to overcome these hurdles (2, 24).

### 5.2 Depalmitoylase inhibitors

An alternative strategy is to inhibit depalmitoylases, effectively “locking” oncoproteins in a palmitoylated state, which can lead to their misfunction, aberrant trafficking, and subsequent degradation. The concept of targeting depalmitoylation in hematologic malignancies was first validated over a decade ago, when palmostatin B, an inhibitor of acyl protein thioesterase 1 (APT1), was shown to selectively reduce the growth of primary murine hematopoietic progenitors expressing oncogenic NrasG12D, while sparing cells with Kras mutations (75). Through hypervariable region swapping experiments, this selectivity was mapped to the hypervariable region of N-Ras, establishing the palmitoylation/depalmitoylation cycle as a key dependency in NRAS-mutant blood cancers. Building on this foundation, the lysosomal depalmitoylase palmitoyl-protein thioesterase 1 (PPT1) has also garnered attention as a therapeutic target. Inhibitors such as DC661 and the clinical-stage compound GNS561 (Ezurpimtrostat) have demonstrated preclinical efficacy in solid tumor models, where they disrupt the palmitoylation cycle of proteins like NRAS and Sprouty 4, leading to attenuated MAPK signaling and re-sensitization to chemotherapy (19). More recently, the cytosolic ABHD17 family of depalmitoylases has emerged as a key regulator of NRAS. The covalent inhibitor ABD957 selectively targets ABHD17 enzymes, impairing NRAS depalmitoylation, disrupting its plasma membrane localization, and synergizing with MEK inhibition to suppress the growth of NRAS-mutant AML cells (76). This represents a highly targeted approach for NRAS-mutant hematologic malignancies, where direct inhibition of the mutant oncoprotein remains challenging. Overall, DEP inhibitors offer a promising therapeutic avenue, with evidence spanning from early genetic and pharmacological validation to current selective inhibitors. Ongoing clinical evaluation of PPT1 inhibitors and continued optimization of ABHD17-targeted agents will be critical for defining their therapeutic utility in blood cancers (19, 77).

### 5.3 Combination therapeutic strategies

Given the complexity of oncogenic and metabolic networks, combining palmitoylation-targeting agents with other therapies offers a rational strategy to enhance efficacy and circumvent resistance. The best-characterized example in hematologic malignancies is the combination of depalmitoylase inhibition with MAPK pathway blockade. The ABHD17 inhibitor ABD957 synergizes with MEK inhibition to suppress NRAS-mutant AML cell growth (76), and recent *in vivo* studies confirm that combining an ABHD17 inhibitor with the MEK inhibitor PD0325901 significantly extends survival in murine Nras-mutant AML models. This synergy extends to other targeted agents, including PI3K, KRASG12C, and FLT3 inhibitors, with evidence of restored gilteritinib sensitivity in a patient-derived xenograft model of adaptive resistance (77). In FLT3-ITD-mutated AML, FASN inhibition with TVB-3166 reduces phospho-Akt and phospho-S6 kinase levels and synergizes with the Gli inhibitor GANT61 to suppress cell survival, supporting the broader concept of combining lipid metabolism inhibitors with targeted therapies (78).

Palmitoylation modulation may also enhance immunotherapy. Given that DHHC3-mediated palmitoylation stabilizes PD-L1 on tumor cells (79) and that palmitoylation regulates immune checkpoint proteins more broadly (17), combining PAT inhibitors with immune checkpoint blockade could reduce PD-L1 surface expression and potentiate T-cell-mediated tumor killing. Although direct evidence in hematologic malignancies is still lacking, this approach holds promise for overcoming immunotherapy resistance. Nanotechnology-based platforms are also being developed to co-deliver such drug combinations, potentially improving tumor targeting and reducing systemic toxicity (80, 81). Together, these emerging combination strategies, supported by both preclinical studies and early *in vivo* validation, point toward more effective and durable therapeutic interventions for hematologic malignancies. In MM, histone deacetylase inhibitors have been shown to enhance CIK-cell cytotoxicity through increased NKG2D-ligand expression, illustrating how post-translational and epigenetic modulation can sensitize malignant plasma cells to cellular immunotherapy (82). A related CIK-cell/PPAR-ligand framework has also been proposed in CNS malignancies; although not direct evidence in blood cancers, it supports cross-tumor investigation of lipid-sensing pathways in combination immunotherapy (83).

## 6 Challenges & future directions

Despite significant progress, translating palmitoylation-targeted therapies into clinical practice faces several well-defined challenges. Technical and biological limitations remain substantial. Current profiling methods (e.g., acyl-biotin exchange) lack the quantitative accuracy and site-specific resolution needed to capture transient, low-abundance palmitoylation events in clinical samples (84–86). The functional redundancy among over 20 ZDHHC enzymes complicates efforts to attribute specific pathological roles to individual PATs and raises concerns about compensatory resistance mechanisms (18, 19). Conversely, oncoproteins like NRAS are modified by multiple PATs and depalmitoylases, creating complex regulatory networks that are difficult to disrupt predictably (12, 76). Moreover, palmitoylation is essential for normal hematopoiesis and immune function (3, 4, 21), making on-target toxicity a major concern that requires careful identification of therapeutic windows (22).

Translational gaps must be bridged through improved preclinical models and predictive biomarkers. Humanized mouse models and patient-derived organoids offer more physiologically relevant platforms for studying palmitoylation in the tumor microenvironment and testing inhibitors (87–89). Patient stratification will require biomarkers, such as proteomic defined “hyper-palmitoylated” subtypes or genetic alterations in specific ZDHHC enzymes linked to pathway dependency (16). Correlating palmitoylation signatures of oncoproteins like NRAS or FLT3-ITD with treatment response could guide personalized combination strategies (84), and AI-driven analysis of multi-omics data may accelerate biomarker discovery (90).

Emerging research avenues offer solutions to these challenges. Decoding the crosstalk between palmitoylation and other post-translational modifications (e.g., phosphorylation, ubiquitination, lactylation) will reveal more nuanced regulatory mechanisms and new therapeutic nodes (91). Nanotechnology-based delivery systems can enable tumor-specific release of PAT/DEP inhibitors, minimizing systemic toxicity (80, 92), while degrader technologies like PROTACs offer a path to selectively eliminate specific PATs or palmitoylated oncoproteins (23, 24). Finally, AI and computational modeling are poised to transform the field by predicting novel palmitoylation sites, identifying non-canonical enzyme-substrate relationships, and modeling system-wide consequences of targeted inhibition (93).

Together, these advances position the field to move from broad inhibition toward context-specific, biomarker-guided precision modulation of palmitoylation, ultimately translating mechanistic insights into transformative therapies for hematologic malignancies (2).

A central limitation of the field is the uneven evidence base. Mechanisms demonstrated directly in AML, APL, lymphoma, or disease-relevant hematopoietic models should be distinguished from findings derived from solid tumors, non-malignant tissues, or pan-cancer datasets. In this review, SCD1-dependent substrate acylation, palmitoylation of PI3K/AKT components, checkpoint regulation outside lymphoma, and several stromal or immune-cell mechanisms are therefore treated as cross-tumor inferences. Lineage identity, membrane composition, metabolic dependence, and treatment exposure may substantially alter palmitoylation phenotypes; consequently, extrapolated mechanisms require direct validation in hematological malignancies.

Palmitoylation also operates within an interconnected post-translational-modification network. By changing membrane localization and protein conformation, palmitoylation can determine access to kinases, phosphatases, ubiquitin ligases, and deubiquitinases. Conversely, phosphorylation may alter cysteine accessibility or regulate ZDHHC/depalmitoylase activity, whereas ubiquitination can cooperate or compete with palmitoylation to control internalization, recycling, proteasomal turnover, and lysosomal degradation (20, 88). Such crosstalk offers a mechanistic basis for adaptive resistance: therapy-induced phosphorylation can redirect protein trafficking, palmitoylation can preserve membrane-proximal survival signaling, and reduced ubiquitin-dependent degradation can stabilize oncogenic or immune-evasion proteins. Mapping these modification combinations over time, rather than measuring palmitoylation in isolation, will be essential for identifying durable therapeutic vulnerabilities. HDAC6 is a particularly relevant example of a non-palmitoylation post-translational regulator at the interface of cancer signaling, proteostasis, and therapeutic development, reinforcing the need to study coordinated modification networks rather than palmitoylation in isolation (94).

## 7 Conclusion

Protein palmitoylation links lipid metabolic reprogramming, oncogenic signaling, and tumor–immune interactions to therapeutic resistance in hematological malignancies. The strongest direct evidence currently concerns selected dependencies in AML and lymphoma, whereas mechanisms in MM, macrophages, MDSCs, stromal cells, and several signaling pathways remain largely inferential. This distinction does not diminish their importance; rather, it defines a focused agenda for cell-type-resolved palmitoyl-proteomics, causal perturbation, and biomarker-guided combination studies. Selective targeting of palmitoylation, particularly when integrated with metabolic modulation and immunotherapy, may provide new opportunities to overcome treatment resistance, provided that antitumor efficacy is balanced against effects on normal hematopoiesis and immune function.
